# Adalimumab in Japanese patients with active ulcers of pyoderma gangrenosum: Final analysis of a 52‐week phase 3 open‐label study

**DOI:** 10.1111/1346-8138.16337

**Published:** 2022-03-03

**Authors:** Kenshi Yamasaki, Keiichi Yamanaka, Yiwei Zhao, Shunsuke Iwano, Keiko Takei, Koji Suzuki, Toshiyuki Yamamoto

**Affiliations:** ^1^ Department of Dermatology Tohoku University Graduate School of Medicine Sendai Japan; ^2^ Department of Dermatology Mie University Graduate School of Medicine Tsu Japan; ^3^ AbbVie Inc. Cambridge Massachusetts USA; ^4^ AbbVie GK Tokyo Japan; ^5^ Department of Dermatology Fukushima Medical University Fukushima Japan

**Keywords:** adalimumab, Japanese patient, pyoderma gangrenosum, skin ulcer, tumor necrosis factor‐α

## Abstract

In this 52‐week, phase 3 open‐label study, efficacy and safety of adalimumab were evaluated in Japanese patients with active ulcers due to pyoderma gangrenosum (PG) during a 26‐week treatment period and another 26‐week extension period. Patients received adalimumab 160 mg at week 0, 80 mg at week 2, and 40 mg every week from week 4. At week 26, 12 of 22 patients (54.5%, *p* < 0.001) achieved the primary efficacy endpoint of PG area reduction 100 (PGAR 100, complete skin re‐epithelialization) for the target ulcer. Nine patients with Physician’s Global Assessment (PGA) score of 1, 2, or 3, including four patients achieving PGAR 100, continued into the extension period. During the extension period, six of nine patients (66.7%) achieved PGAR 100 for the target PG ulcer at 52 weeks; one patient who achieved PGAR 100 before week 26 experienced a relapse 162 days after achieving this endpoint. Six patients achieved PGA 0 by week 52, and one patient reported new ulcers at day 57 of the extension period. Continued improvements from study baseline to week 52 were observed in pain (mean [95% CI] –4.0 [−6.5 to −1.5] numeric rating scale) and Dermatology Life Quality Index (−7.3 [−15.1 to 0.4]). In addition to the adverse events (AE) reported in 18 patients (including four serious AE) through week 26 (most commonly infections [*n* = 11]), there was one 1 additional AE (infection) during the extension period. These results suggest that adalimumab is effective and generally well tolerated in Japanese patients with active PG ulcers.

## 1 INTRODUCTION

Pyoderma gangrenosum (PG) is a rare, chronic, inflammatory skin disease characterized by nodules and pustules that develop into deep, painful ulcers.^1^, ^2^ First‐line therapy is commonly systemic corticosteroids or cyclosporine.^3^, ^4^ Biologics, such as the tumor necrosis factor (TNF) inhibitors, infliximab and adalimumab, have been used successfully in the treatment of PG ulcers, predominantly in case studies,^5^, ^6^, ^7^, ^8^, ^9^ in a small placebo‐controlled trial,^10^ and in two retrospective studies.^11^, ^12^ The interleukin (IL)‐23 inhibitor, risankizumab, the IL‐12/23 inhibitor ustekinumab, and the IL‐17 inhibitor brodalumab have also been reported to be successful in a small number of case reports.^13^, ^14^, ^15^

To address the need for alternative medication for refractory PG, we carried out a phase 3 randomized open‐label study to evaluate the efficacy, safety, and pharmacokinetics of adalimumab 40 mg every week for 52 weeks in Japanese patients with active ulcers of PG. Analysis at 26 weeks showed that the primary endpoint of PG area reduction 100 (PGAR 100, defined as complete skin re‐epithelialization) in the target ulcer was achieved by 12 of the 22 participants (54.5%).^16^ Findings from this interim analysis supported the approval of adalimumab (Humira®) for the treatment of PG in Japan in November 2020.

We now report secondary per‐protocol and other endpoints for the target PG ulcer and for PG ulcers overall in patients who had achieved some improvement in their PG ulcers, but not yet complete resolution at 26 weeks, and who continued adalimumab treatment to 52 weeks.

## 2 METHODS

### 2.1 Study design and participants

**FIGURE 1:**
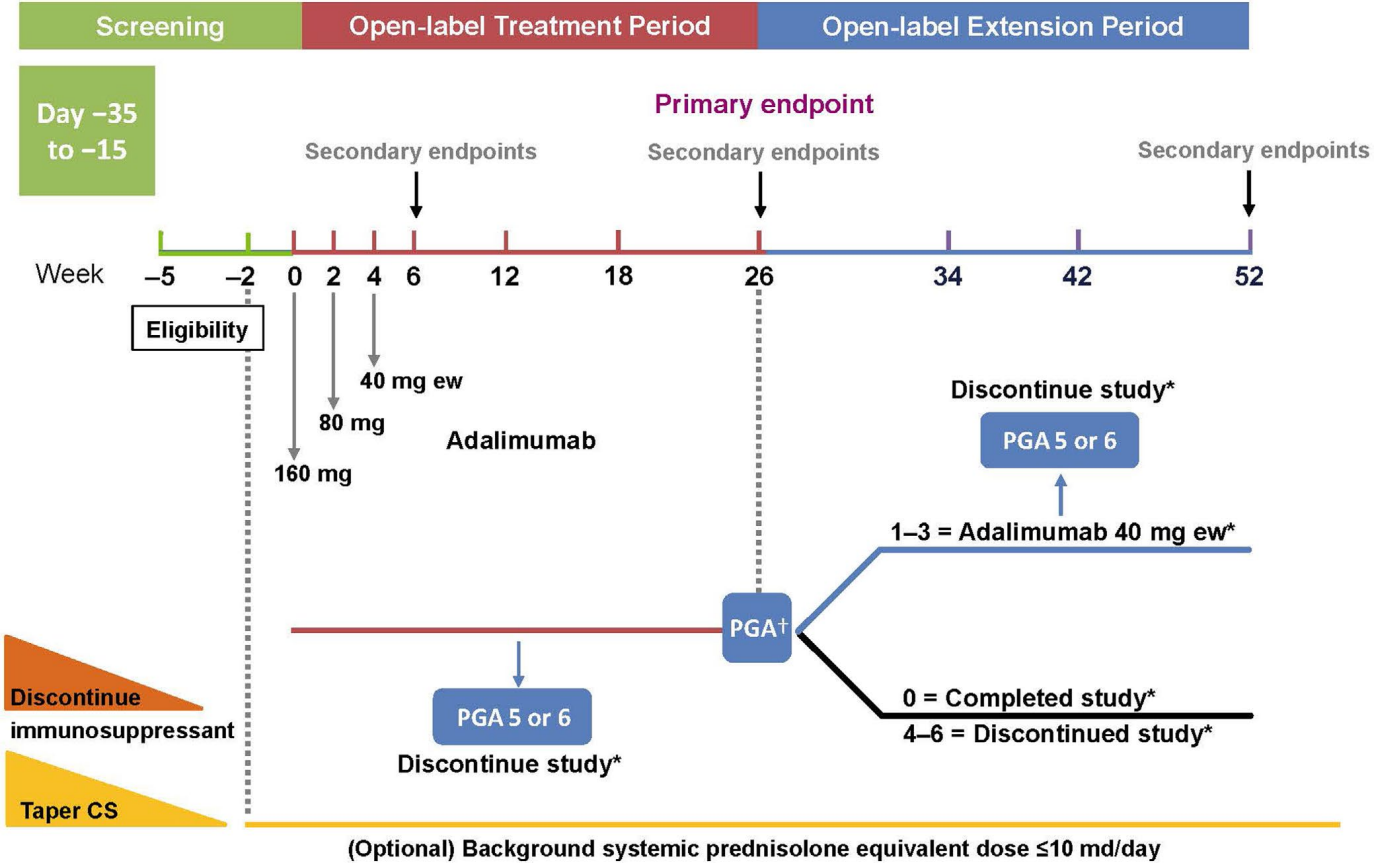
Study design. ^†^Follow up: Patients were contacted approximately 70 days following study drug discontinuation for an assessment of any new or ongoing adverse effects (AE). ^‡^Patients who reached improvement of ulcers with a PGA score of 1, 2, or 3 at week 26, could enter the extension period to receive adalimumab 40 mg every week until week 52. Patients who achieved healing of all ulcers (a PGA score of 0) at week 26 were considered to have completed the study, and patients who had a PGA score of 4–6 at week 26 were discontinued from the study. CS, corticosteroid; ew, every week; PGA, Physician’s Global Assessment

This phase 3, open‐label, single arm, multicenter study enrolled patients at 15 study sites in Japan. After a screening period of up to 5 weeks, patients with active PG ulcers (defined as ulcers with a score of ≥1, on the two 5‐point scales of erythema and border elevation of the Investigator’s Inflammation Assessment, in which 0 = none and 4 = very severe; Table S1)^17^ entered a 26‐week treatment period during which they received adalimumab 160 mg s.c. at week 0, followed by 80 mg at week 2 and 40 mg every week starting at week 4 (Figure 1).

Patients who achieved healing of all ulcers (Physician’s Global Assessment [PGA] score of 0 [completely clear]; Table S2) at week 26 were considered to have completed the study; patients who had a PGA score of 4, 5, or 6 (slight improvement, no change, or worse) at week 26 were discontinued from the study. Patients, who had a PGA score of 1, 2, or 3 (almost clear, marked improvement, or moderate improvement) at week 26 were included in a 26‐week extension period during which they received adalimumab 40 mg every week until week 52. Results for these patients are reported here.

### 2.2 Inclusion/exclusion criteria

Eligibility criteria for the study have been reported previously.^16^ In brief, patients ≥18 years old, diagnosed by the investigator as having active ulcerative (classic) PG (including peristomal PG), and who had an inadequate response to or were not candidates for topical PG therapy, were eligible for inclusion. Patients were required to have at least one active and measurable ulcer with a distinct margin surrounded by epithelialized skin. Target PG ulcers were ≥3 to <10 cm if non‐peristomal, or ≥1 to <10 cm if peristomal (excluding stoma) in their largest dimension at week −2. Patients were excluded if they had received topical treatment for PG within 14 days prior to baseline, had been previously exposed to adalimumab, were receiving a biologic agent or systemic treatment for PG, or had discontinued biologics or systemic treatments (such as cyclosporine, mycophenolic acid, azathioprine, diaphenylsulfone/dapsone, i.v. immunoglobulin) within five half‐lives of each drug prior to week −2.

Patients receiving systemic corticosteroids at screening were required to taper the dose to ≤10 mg prednisolone equivalent at week −2, and to maintain this dose until the end of the study (except if an adverse event [AE] related to corticosteroid use was suspected). Patients treated with immunosuppressants for PG at screening were excluded; immunosuppressants (including azathioprine/6‐MP, sulfasalazine or salazosulfapyridine, mesalazine, methotrexate, or leflunomide) were permitted for the treatment of comorbidities only (e.g., inflammatory bowel disease, rheumatoid arthritis) and were maintained at the same dose until the end of the study (except if an AE related to drug was suspected). The study was conducted in accordance with the protocol, International Conference on Harmonization guidelines, applicable regulations, and the ethical principles of the Declaration of Helsinki. All patients reviewed and signed an informed consent prior to any study procedures. An independent ethics committee or institutional review board at each study site approved the study protocol, informed consent form, and other study‐related documents.

### 2.3 Study endpoints

The primary efficacy endpoint of this study, the proportion of patients who achieved PG area reduction (PGAR) 100 (defined as complete skin re‐epithelialization) for the target ulcer at week 26 as assessed by photography‐based digital measurements (digital planimetry) (Table S3) as well as the key secondary endpoints at week 26, have been described previously.^16^

#### 2.3.1 Secondary and other endpoints for the open‐label extension period

Secondary and other endpoints for the extension period were evaluated in the nine patients who entered and completed the extension period of the study. This included all patients who received at least one dose of study drug and had at least one post‐treatment efficacy assessment during the extension period. Target PG ulcer endpoints included the proportion of patients achieving PGAR 100 at 52 weeks of adalimumab treatment; mean time to healing (PGAR 100) as assessed by the patient, estimated to the nearest week and captured by the investigator using digital planimetry at the first opportunity (scheduled or unscheduled visit) through week 52; mean time to relapse of the target PG ulcer (recurrence, PGAR < 100) when PGAR 100 had been achieved prior to week 52; and percentage change in target PG ulcer area (by digital planimetry).

In addition, the following key secondary and other endpoints at 52 weeks were assessed to evaluate global improvement in patients (all PG ulcers, including the target PG ulcer): proportion of patients achieving PGA 0; mean time to occurrence of new PG ulcers (defined as an ulcer not present at baseline and not caused by epithelial bridging of an ulcer present at baseline); proportion of patients achieving PGA 0 or 1; change from baseline in pain evaluated using a Numeric Rating Scale (NRS); and change from baseline in Dermatology Life Quality Index (DLQI).

#### 2.3.2 Safety

Adverse events, laboratory data, physical examinations, and vital signs were monitored at designated study visits. AE, serious AE, AE leading to discontinuation, and prespecified AE of special interest were collected up to approximately 70 days after the last dose of study drug. Numbers and percentages of patients experiencing AE were tabulated using the Medical Dictionary for Drug Regulatory Activities (MedDRA® version 22.0) system organ class and preferred term. A listing of all patients with any laboratory determination meeting Common Toxicity Criteria (CTC version 4.0) of Grade 2 or higher was prepared.

### 2.4 Statistical analyses

The safety population for the 52‐week analysis included all patients who entered the extension phase of the study and received at least one dose of study drug (*n* = 9). Missing data were imputed using non‐responder imputation (NRI) and last observation carried forward (LOCF) in patients completing the extension period.

For binary endpoints, frequencies, percentages, and 95% confidence interval (CI) were reported; mean, standard deviation (SD), median and range, and 95% CI were reported for continuous endpoints. Analyses were performed using SAS version 9.4® (SAS Institute ).

## 3 RESULTS

### 3.1 Study population

**FIGURE 2:**
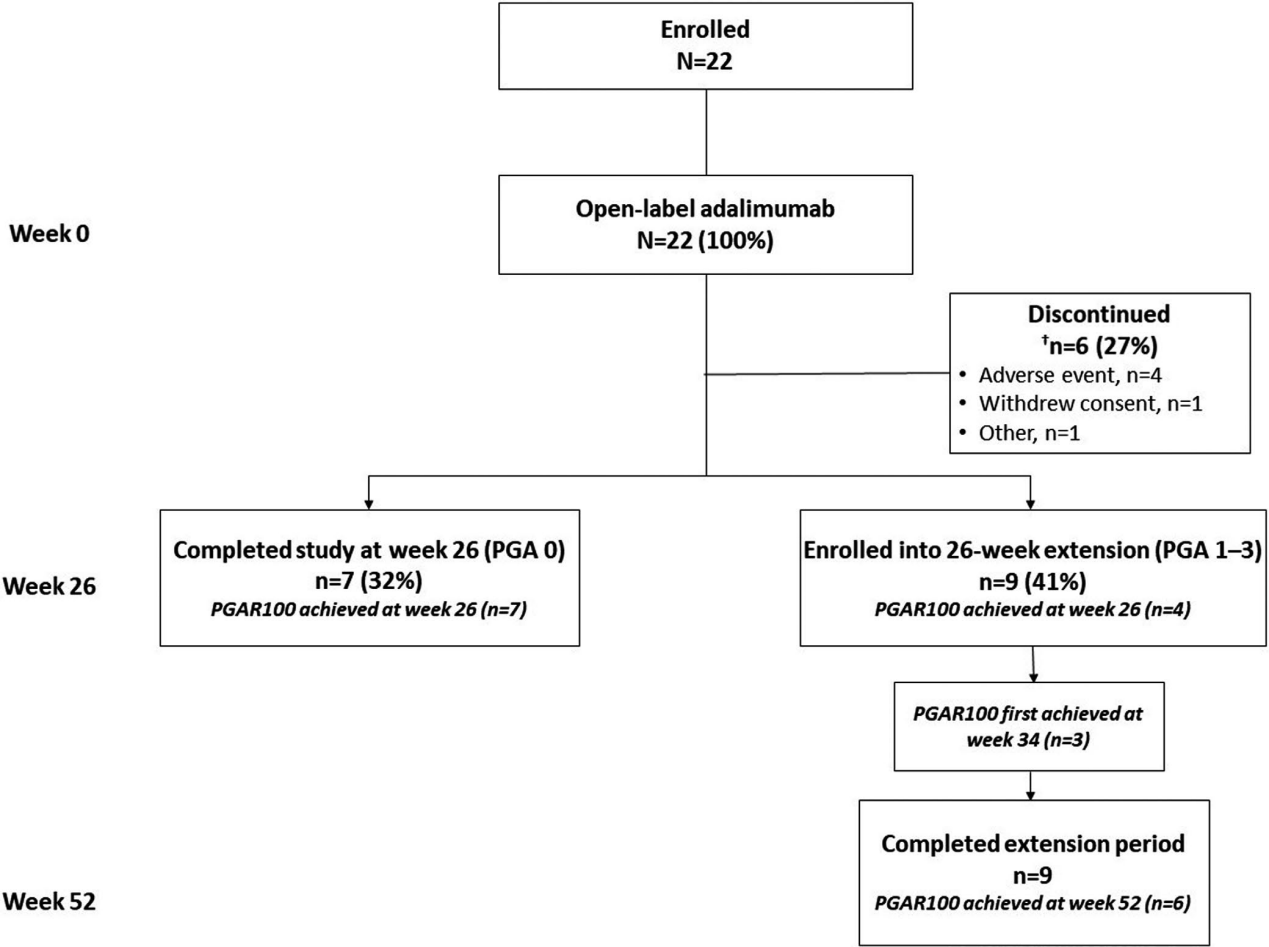
Patient disposition. ^†^One patient who achieved PGA 0 and PGAR 100 in the 26‐week analysis discontinued the study. PGA, Physician’s Global Assessment of all ulcers; PGAR 100, complete re‐epithelialization of target ulcer

Twenty‐two Japanese patients were enrolled,^16^ of whom eight (36%) achieved PGA 0 at week 26 assessment. Seven of these patients completed the study at week 26; one patient discontinued after providing data that were included in the 26‐week analysis. In total, six patients discontinued during the 26‐week treatment period (four due to an AE, one withdrew consent, one other reasons).^16^ The remaining nine patients (41%), with PGA scores of 1–3, continued into the 26‐week extension period and all completed the study at week 52 (Figure 2). No patients had PGA scores of 4–6 at week 26.

### 3.2 Demographics

Baseline demographics for the study population (*n* = 22) and those enrolled into the extension study (*n* = 9) are given in Table 1.^16^ At the start of the study, mean age was 56.4 years (range, 21–83); 12 (55%) patients were female. Target PG ulcer mean area was 33.3 cm^2^ (by digital planimetry) at baseline, and the target PG ulcers were mostly located on a leg (18/22, 82%). Mean duration of PG was 3.3 years. Baseline comorbidities included hypertension (45%), hyperlipidemia (27%), hyperuricemia (23%), osteoporosis and ulcerative colitis (18% each), and rheumatoid arthritis (14%); one patient presented with peristomal dermatitis. Mean pain NRS was 4.6 and DLQI was 9.3. Sixteen patients (73%) had concomitant corticosteroid use at baseline, including 13 (59%) who used corticosteroids for the treatment of PG (Table 1). One patient had previously received etanercept for PG.

**TABLE 1:** Baseline demographics and disease characteristics

	Open‐label treatment period, *n* = 22	Open‐label extension period, *n* = 9
Female, *n* (%)	12 (55)	5 (5.6)
Age, y		
Mean (SD)	56.4 (18.6)	60.7 (17.3)
Median (min, max)	60.5 (21.0, 83.0)	62.0 (29.0, 81.0)
BMI, mean (SD), kg/m^2^	25.5 (7.3)	29.2 (9.5)
Disease duration, mean (SD), y	3.3 (5.3)	4.6 (7.1)
Comorbidities, *n* (%)		
Hypertension	10 (45)	6 (67)
Hyperlipidemia	6 (27)	2 (22)
Hyperuricemia	5 (23)	4 (44)
Osteoporosis	4 (18)	2 (22)
Ulcerative colitis	4 (18)	1 (11)
Rheumatoid arthritis	3 (14)	1 (11)
Target PG ulcer area (digital planimetry), mean (SD), cm^2^	33.3 (25.2)	32.4 (20.9)
IIA, moderate to very severe, *n* (%)		
Erythema	16 (73)	6 (67)
Border elevation	18 (68)	7 (78)
Pain; NRS, mean (SD)	4.6 (3)	5.0 (3)
DLQI, mean (SD)	9.3 (7)	10.2 (8)
Baseline corticosteroid use, *n* (%)	16 (73)	6 (67)
Dose 10 mg/day, *n* (%)	13 (59)	5 (56)
Dose <10 mg/day, *n* (%)	3 (14)	1 (11)
Corticosteroid use for PG, *n* (%)	13 (59)	5 (56)

Abbreviations: BMI, body mass index; DLQI, Dermatology Life Quality Index; IIA, investigator inflammation assessment; NRS, numeric rating scale; PG, pyoderma gangrenosum; SD, standard deviation.

Demographic and baseline clinical characteristics of the nine patients who had a partial response (PGA 1, 2, or 3) at week 26 of the treatment period and who continued into the extension period of the study were broadly similar to those of the overall patient population who commenced the study (Table 1 and Table S4).

### 3.3 Target ulcer improvement endpoints

**FIGURE 3:**
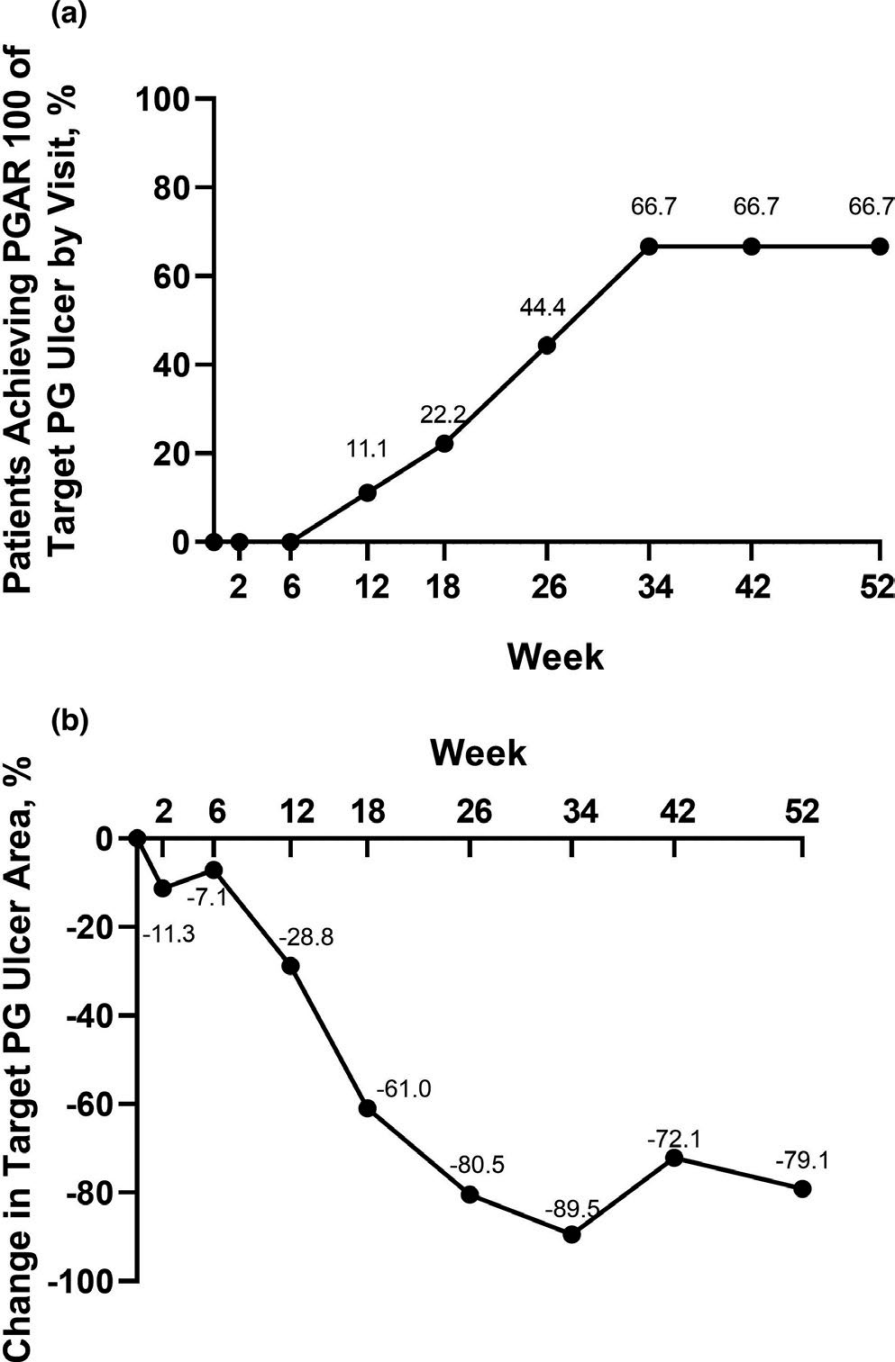
(a) Proportion of patients achieving PGAR 100 for the target PG ulcer by visit (NRI) and (b) percentage change in target PG ulcer area by digital planimetry (LOCF; *n* = 9). LOCF, last observation carried forward; NRI, non‐responder imputation; PG, pyoderma gangrenosum; PGAR 100, target PG ulcer area reduction 100 (defined as complete skin re‐epithelialization)

Of the nine patients continuing into the extension period, four (44.4%) patients achieved a PGAR 100 (complete skin re‐epithelialization) for the target PG ulcer at week 26. A total of six of nine (66.7%) patients achieved PGAR 100 for the target PG ulcer at 52 weeks (Figure 3a). Time (days) to healing of the target ulcer through week 52 was calculated using time of entry into the extension period as the start time; any patients who had already achieved PGAR 100 at 26 weeks were excluded from this calculation. Mean (SD) time to healing (PGAR 100) of the target PG ulcer through week 52 was 74.0 (28.4) days (median [range], 64 (52–106) days [*n* = 3]). Figure S1 shows per visit progress of the target PG ulcers for these three patients. One patient who achieved PGAR 100 prior to, or at, week 26 experienced a relapse of the target ulcer (recurrence) 162 days after achieving this endpoint. Percentage change from baseline in target PG ulcer area during the 52 weeks of the study indicates that healing was most rapid during weeks 12–34 (Figure 3b).

### 3.4 Global improvement endpoints

**FIGURE 4:**
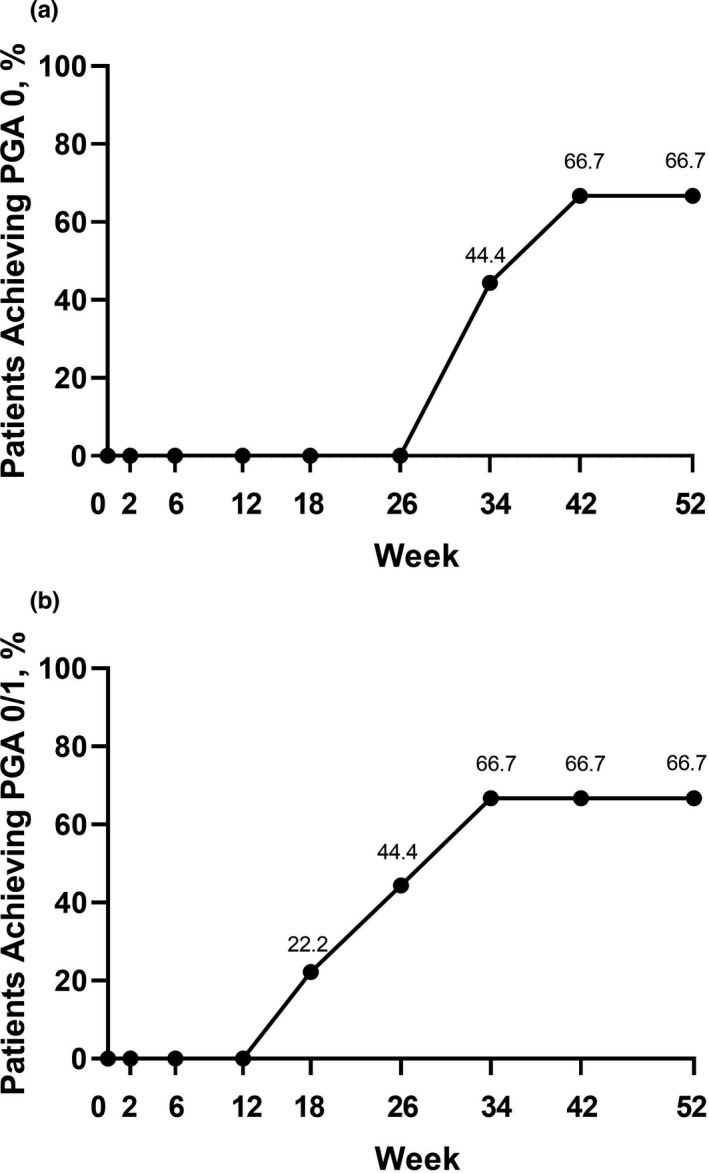
Proportion of patients achieving (a) PGA 0 for all PG ulcers and (b) PGA 0 or 1 for all PG ulcers (patients in the extension period, NRI; *n* = 9). NRI, non‐responder imputation; PG, pyoderma gangrenosum; PGA, Physician’s Global Assessment

**FIGURE 5:**
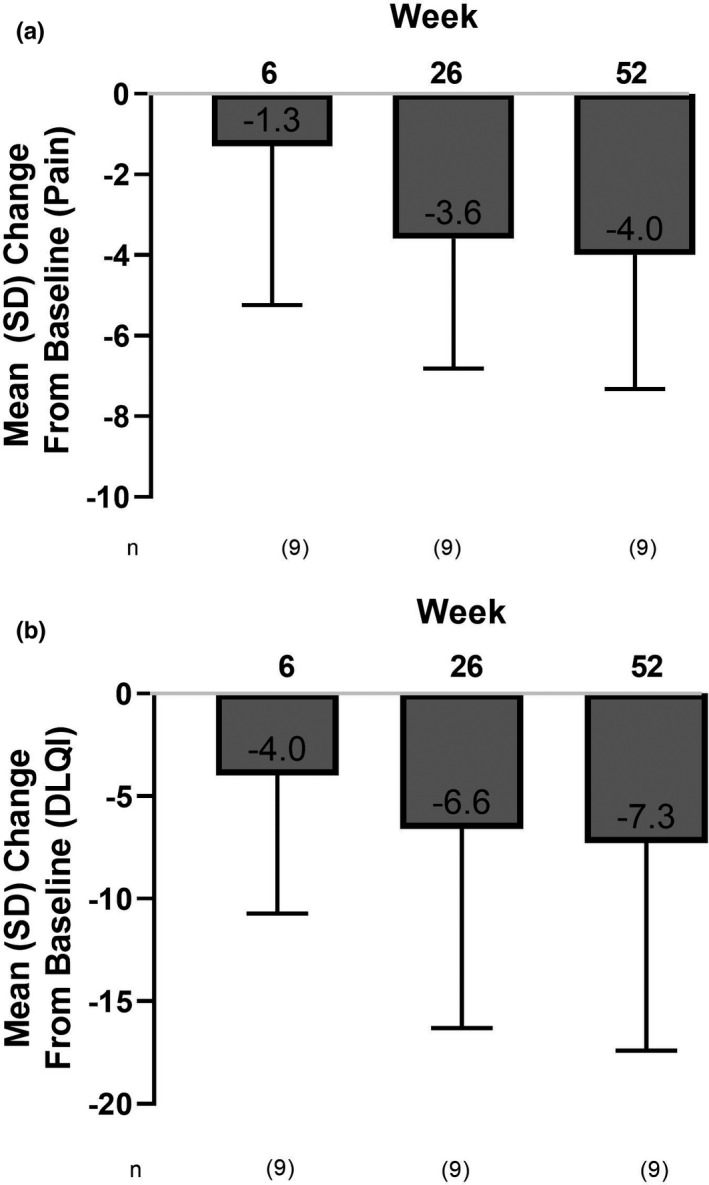
Mean change from baseline in (a) patient pain NRS and (b) DLQI at weeks 6, 26, and 52. DLQI, Dermatology Life Quality Index; NRS, numeric rating scale

All PG ulcers, including the target PG ulcer, were assessed for global improvement endpoints. Six of the nine patients (67%) who achieved PGA 1, 2, or 3 during the 26‐week treatment period and entered the extension period, achieved PGA 0 by week 52, four of them achieved this endpoint by week 34 (Figure 4a). Seven patients reported the development of new PG ulcers during the initial 26‐week treatment period, with a mean time to occurrence of new ulcers of 22.1 days.^16^ During the extension period, one patient reported new ulcers, with mean time to occurrence of 57 days from the start of the extension period. By week 34, six patients had achieved PGA 0 or 1 (Figure 4b **)**. In patients who entered the extension period, mean (95% CI) pain NRS (LOCF) was significantly improved from baseline at week 26 (−3.6 [−6.0 to −1.1]) and at week 52 of the study (−4.0 [−6.5 to −1.5]). (Figure 5a). Patients who entered the extension period, experienced a numeric improvement in mean (95% CI) DLQI compared with baseline at week 6 (−4.0 [−9.2 to 1.2]). Further improvements were reported at the end of the 26‐week treatment period −6.6 [−14.0 to 0.9]) and at the end of the extension period (−7.3 [−15.1 to 0.4]) (Figure 5b).

### 3.5 Safety

Safety data for the entire 52‐week study are shown in (Table 2). In addition to AE reported through week 26, there was only one additional AE (infection) among the nine patients in the extension period. Safety data for the initial 26‐week treatment period have been presented previously.^16^ Briefly, during the 26‐week treatment period, 18 (82%) patients experienced at least one treatment‐emergent AE, leading to study drug discontinuation in four (18%) patients (Table 2). Serious AE were reported in four (18%) patients (one each of anemia, bacterial arthritis [led to discontinuation of study drug], cataract, and pain due to PG). Most AE were mild or moderate in severity. AE reported most frequently (≥10% of patients) were nasopharyngitis (five incidences in four patients), anemia, Cushingoid, eczema, and insomnia (three patients each), and PG (reported name: aggravated PG in one patient, worsening of PG in one patient, and pain attributed to PG in one patient). There were no events of opportunistic infection, tuberculosis (active or latent), progressive multifocal leukoencephalopathy, malignancy, intestinal perforation, or cardiopulmonary‐ or liver‐related AE. There was one death during the screening period (due to acute ascending aortic dissection).^16^

**TABLE 2:** Treatment‐emergent adverse events reported during the 26‐week treatment period and the 52‐week study

Treatment‐emergent AE *n* (%)	26‐week treatment period^a^, *n* = 22	52‐week study^b^, *n* = 22
Any AE	18 (81.8)	18 (81.8)
Severe AE	2 (9.1)	2 (9.1)
Serious AE	4 (18.2)	4 (18.2)
AE leading to discontinuation of study drug	4 (18.2)	4 (18.2)
Serious AE leading to discontinuation of study drug	0	0
AE possibly related to study drug	9 (40.9)	9 (40.9)
Serious AE possibly related to study drug	2 (9.1)	2 (9.1)
AE of special interest		
Infection	11 (50.0)	12 (54.5)
Serious infection	1 (4.5)	1 (4.5)
Opportunistic infections	0	0
Oral candidiasis	0	0
Tuberculosis	0	0
Malignancy	0	0
Allergic reaction (including angioedema/anaphylaxis)	2 (9.1)	2 (9.1)
Injection‐site reaction	1 (4.5)	1 (4.5)
Deaths	0	0

Abbreviations: AE, adverse event.

^a^ Data cutoff date 20 August 2019.

^b^ Data cutoff date 24 June 2020.

## 4 DISCUSSION

In this report, we describe outcomes for the secondary per‐protocol and other endpoints for all patients, including nine patients (41%) who achieved PGA scores of 1–3 for all PG ulcers at week 26 and entered the 26‐week extension period, providing further long‐term data on the efficacy and safety of adalimumab in this patient population.

At week 26 of adalimumab treatment, 12 (54.5%) patients had achieved the primary study endpoint of PGAR 100 for the target PG ulcer, including eight patients who achieved PGA 0 and completed the study (one discontinued) and four patients who achieved PGA 0/1 and entered the extension. Of the five patients who had not achieved PGAR 100 at 26 weeks, three of them did so by 34 weeks.

Mean (SD) time to healing of the target ulcer (PGAR 100) during the extension period was 74.0 (28.3) days (*n* = 3); in one patient, healing occurred after only 52 days. Progress of the healing of target PG ulcers throughout the 52‐week study for these three patients are shown in Figure S1. One patient who had achieved PGAR 100 by week 26 experienced recurrence of the target ulcer at day 162 of the extension period.

Global improvement endpoints at week 26,^16^ showed that during the treatment period there was a continuous decrease in the total ulcers area. PGA 0 was achieved by two patients (9.1%) at week 6 and eight patients (36%) at week 26, while PGA 0/1 (completely/almost clear) was achieved by five (22.7%) and 12 patients (54.5%) at weeks 6 and 26, respectively. During the extension period, six of the nine patients (67%) who achieved PGA 1, 2, or 3 during the 26‐week treatment period and entered the extension period achieved PGA 0 by week 52 (four patients achieved this endpoint by week 34), demonstrating that extended treatment with adalimumab is beneficial in patients who show a response to adalimumab but have not achieved complete healing of all PG ulcers by week 26.

Frequency of new PG ulcers was low; seven patients reported the development of new PG ulcers during the 26‐week treatment period (mean time to occurrence, 22.1 days).^16^ Five of these patients continued treatment with adalimumab at the same dose, and the new ulcers subsequently resolved; the remaining two patients discontinued treatment. As expected, among such a small number of patients, comparison of the baseline demographic and clinical characteristics of patients who developed new PG ulcers and those who did not, yielded few insights: a higher proportion of patients developing new ulcers were female (six out of seven) compared with the overall study population (86% vs. 55%), and one of the four patients with ulcerative colitis and two of the three patients with rheumatoid arthritis at baseline were among those who developed new PG ulcers. Only one of the nine patients who continued treatment to 52 weeks reported new PG ulcers (57 days after the start of the extension period).

The proportion of patients achieving PGAR 100 for the target ulcer at 26 weeks of adalimumab treatment (54.5%) is similar to that in the earlier STOP GAP randomized controlled trial, in which 47% of patients treated with either cyclosporine or prednisolone achieved complete re‐epithelialization of the target ulcer within 6 months.^18^ However, it should be noted that the definitions of “complete healing” were not identical between these two trials: in STOP GAP, complete healing was defined as an ulcer no longer requiring dressings, whereas we defined PGAR 100 as complete closure of the target PG ulcer.

Results from the 26‐week extension period in our study indicate that patients who show an initial response to treatment may benefit from a further period of treatment with adalimumab; three additional patients who achieved PGA 1–3 during the initial 26 weeks achieved PGAR 100 by 52 weeks.

As reported,^16^ safety during the 26‐week treatment period was consistent with the known safety profile of adalimumab, with no new or unexpected AE reported. Among the nine patients who entered the extension period there was one additional AE (an infection, cause unrecorded). Our data thus support longer term (up to 1 year) efficacy and safety of weekly adalimumab in the treatment of PG. Although enrolling a small number of patients (a consequence of the low prevalence of PG), in both the initial treatment period (*n* = 22) and the extension period (*n* = 9), this study represents the single largest collection of cases of confirmed active ulcers of PG treated with adalimumab for up to 1 year reported to date.

Broader interpretation of these data is limited by the small number of patients (*n* = 22). Indeed, to date, only a small number of randomized clinical trials of systemic treatment of PG have been reported, and these have used different primary endpoints, including patient/clinician determined clinical improvement, speed of healing of a target lesion, and in our study the more objective measurement PGAR 100.^10^, ^16^, ^18^ Among seven clinical trials included in a systematic review, 20 different PG outcome instruments were used, including 11 physician‐reported instruments, eight patient‐reported instruments, and one composite instrument. Only three (15%) of these (speed of healing, PGA, and resolution of inflammation) had validation data, but were lacking around half of the 2018 COSMIN Risk of Bias checklist categories, leading the authors to conclude that PG validation studies are needed for existing instruments, and that development of a core outcome set for PG additional instruments are required.^19^ Additional limitations of this study are the open‐label single‐treatment design, and the lack of a placebo‐control group; however, recruitment of patients with this painful and disabling condition to a placebo‐controlled trial would raise both ethical and practical considerations.

In conclusion, this study in Japanese patients with confirmed active ulcers of PG, demonstrates that weekly adalimumab 40 mg is beneficial, leading to complete skin re‐epithelialization of the target PG ulcer within 26 weeks in 12 (54.5%) patients^16^ and by 52 weeks in a further two responders with a PGA of 1–3 at 26 weeks, confirming the safety and utility of adalimumab in PG.

## CONFLICT OF INTEREST

Kenshi Yamasaki has served as clinical trials investigator for AbbVie, Amgen/Celgene Boehringer Ingelheim; Japan Eli Lilly Ltd., Kyowa Kirin Ltd., LEO Pharma, Janssen Pharma, Maruho Ltd., MSD, Parexel, and UCB Japan; has received research funding (funding to his department) from Eisai Ltd., Kyowa Kirin Ltd., Maruho Ltd., Shionogi Seiyaku Ltd., Taiho Yakuhin Ltd., Tanabe Mitsubishi Ltd., Tokiwa Yakuhin Kogyo Ltd., and Torii Yakuhin Ltd.; has received research funding from Japan Eli Lilly Ltd., Kao Ltd., and Novartis Pharma; has carried out collaborative research with Allergan, Daiichi‐Sankyo Ltd., Kao Ltd., Maruho Ltd., Teikoku Yakuhin Ltd., and Tsumura Ltd.; has acted as a consultant for AbbVie, Asahi‐Kasei Ltd., Boehringer Ingelheim, Galderma, Janssen Pharma, Japan Eli Lilly Ltd., LEO Pharma, Maruho Ltd., Nihon L’Oreal K, Pfizer Japan Inc., Pola Parma Ltd., and UCB Japan; has received honoraria from AbbVie, Astellas Seiyaku Ltd., Celgene, Chugai Seiyaku Ltd., Cracie Yakuhin Ltd., Daiichi‐Sankyo Ltd., Eisai Ltd., Galderma, Glaxo‐SmithKline, Janssen Pharma, Japan Eli Lilly Ltd., Kyowa‐Hakko Kirin Ltd., LEO Pharma, Maruho Ltd., Mochida Seiyaku Ltd., MSD, Nippon Kayaku Ltd., Nippon Zouki Ltd., Novartis Pharma, Pola Parma Ltd., Rhoto Seiyaku Ltd., Sato Seiyaku Ltd., Sanofi, Shionogi Seiyaku Ltd., Shiseido Ltd, Taiho Yakuhin Ltd., Tanabe Mitsubishi Ltd., Tokiwa Yakuhin Kogyo Ltd., and Torii Yakuhin Ltd. Keiichi Yamanaka has received research grants, speaker’s fees, and chair’s fees from AbbVie. Inc. Yiwei Zhao, Shunsuke Iwano, and Keiko Takei are full‐time salaried employees of AbbVie. Koji Suzuki is a former employee of AbbVie. Toshiyuki Yamamoto has received speaker fees from AbbVie.

## Supporting information


Supinfo

## Data Availability

AbbVie is committed to responsible data sharing regarding the clinical trials we sponsor. This includes access to anonymized, individual and trial‐level data (analysis data sets), as well as other information (e.g., protocols and clinical study reports), as long as the trials are not part of an ongoing or planned regulatory submission. This includes requests for clinical trial data for unlicensed products and indications. These clinical trial data can be requested by any qualified researchers who engage in rigorous, independent scientific research, and will be provided following review and approval of a research proposal and Statistical Analysis Plan (SAP) and execution of a Data Sharing Agreement (DSA). Data requests can be submitted at any time and the data will be accessible for 12 months, with possible extensions considered. For more information on the process, or to submit a request, visit the following link: https://www.abbvie.com/our‐science/clinical‐trials/clinical‐trials‐data‐and‐information‐sharing/data‐and‐information‐sharing‐with‐qualified‐researchers.html.
