# Supplementary material for: Adalimumab in Japanese patients with active ulcers of pyoderma gangrenosum: Final analysis of a 52‐week phase 3 open‐label study
Source: J Dermatol. 2022 Mar 3;49(5):479–87. doi: 10.1111/1346-8138.16337 (PMC9313851; doi:10.1111/1346-8138.16337)
Supplement: Supplementary file 1 — Supinfo [file JDE-49-479-s001.pdf]

Adalimumab in Japanese Patients with Active Ulcers of Pyoderma Gangrenosum:

Final Analysis of a 52-Week Phase 3 Open-label Study

Kenshi Yamasaki, Keiichi Yamanaka, Yiwei Zhao, Shunsuke Iwano, Keiko Takei, Koji Suzuki, Toshiyuki Yamamoto

Supplementary Material

Table S1. Investigator Inflammation Assessment (From Wilkes *et al.* 2016, *J Am Acad Dermatol* http://dx.doi.org/10.1016/j.jad.2016.07.049)

**Severity rating score**		**Description**
**Erythema**		
0	None	No erythema
1	Slight	Mild pink color
2	Moderate	Moderate pink color
3	Severe	Reddish color
4	Very severe	Dark red or violaceous
**Border elevation**		
0	None	Border is flat with ulcer and surrounding skin, no elevation
1	Slight	Slight elevation of border above ulceration and surrounding skin
2	Moderate	Noticeable elevation of border above ulceration and surrounding skin
3	Severe	Significant elevation of border above ulceration and surrounding skin
4	Very severe	Border rolled high above ulceration and surrounding skin

Table S2 Physician's Global Assessment Scale. (From Wilkes *et al.* 2016, *J Am Acad Dermatol* http://dx.doi.org/10.1016/j.jad.2016.07.049)

Scale	Description	
**0**	Completely clear	Except for possible residual hyperpigmentation
**1**	Almost clear	Very significant clearance (about 90%); however, patchy remnants of dusky erythema and/or very small ulceration
**2**	Marked improvement	Significant improvement (about 75%); however, a small amount of disease remaining (ie, remaining ulcers, although have decreased in size, minimal erythema, and/or barely perceptible bolder elevation)
**3**	Moderate improvement	Intermediate between slight and marked; representing about 50% improvement
**4**	Slight improvement	Some improvement (about 25%); however, significant disease remaining (ie, remaining ulcers with only minor decrease in size, erythema, or border elevation)
**5**	No change from baseline	
**6**	Worse	

Table S3. Specification, measurement, and PGAR calculation of target ulcer

1a	Examination of all ulcers at the screening visit
1b	Specification of the target pyoderma gangrenosum (PG) ulcer; this does not change throughout the study<br><br>In the case of multiple PG ulcers, the target PG ulcer is the largest ulcer by area that can be photographed on a single plane
3	Photography of the target PG ulcer and all non-target PG ulcer(s) at specified visits
4a	Using digital photography of a patient's target PG ulcer, borders (and internal epithelialized regions, if any) delineated, and ulcer area calculated (cm^2) using computerized planimetry (by external vendor)
4b	Maximal width and height of the target PG ulcer and all non-target PG ulcers measured by the Investigator at specified visits
5	The PGAR (PG area reduction) calculated as the percentage area change in the target PG ulcer from baseline by digital planimetry
PGAR 100	Pyoderma gangrenosum area reduction 100% (complete skin re-epithelialization)

Table S4. Baseline demographics and disease characteristics of responders (achieving PGAR 100) and partial/non-responders at 26 weeks and 52 weeks

	26 Weeks		52 Weeks	
	Responders n=12	Partial/non-responders† n=10	Responders n=6	Partial/non-responders n=3
Female, n (%)	5 (41.7)	7 (70.0)	4 (66.7)	1 (33.3)
Age, mean (SD), y	48.6 (17.8)	65.7 (15.6)	56.5 (19.0)	69.0 (11.5)
BMI, mean (SD), kg/m^2	25.2 (8.2)	25.9 (6.4)	29.0 (11.5)	29.6 (5.0)
Disease duration, mean (SD), y	4.8 (6.6)	1.5 (2.0)	5.5 (8.4)	2.6 (3.9)
Comorbidities, n (%)				
Hypertension	4 (33.3)	6 (60.0)	5 (83.3)	1 (33.3)
Hyperlipidemia	2 (16.7)	4 (40.0)	1 (16.7)	1 (33.3)
Hyperuricemia	2 (16.7)	3 (30.0)	2 (33.3)	2 (66.7)
Osteoporosis	0	4 (40.0)	1 (16.7)	1 (33.3)
Ulcerative colitis	3 (25.0)	1 (10.0)	1 (16.7)	0
Rheumatoid arthritis	1 (8.3)	2 (20.0)	0	1 (33.3)
Target PG ulcer area (digital planimetry), mean (SD), cm^2	29.8 (25.2)	37.5 (25.8)	36.7 (22.3)	24.0 (18.7)
IIA, moderate to very severe, n (%)				
Erythema	10 (83.3)	6 (60.0)	4 (66.7)	2 (66.7)
Border elevation	8 (66.7)	7 (70.0)	5 (83.3)	2 (66.7)
Pain; NRS, mean (SD)	4.2 (3.2)	5.2 (2.5)	5.5 (3.3)	4.0 (1.0)
DLQI, mean (SD)	9.4 (8.5)	9.1 (5.8)	12.7 (9.4)	5.3 (3.5)
Corticosteroid use for PG, n (%)‡	8/11 (72.7)	5/5 (100%)	4/5 (80.0)	1/1 (100)

BMI, body mass index; DLQI, Dermatology Life Quality Index; IIA, investigator inflammation assessment; NRS, numeric rating scale; PG, pyoderma gangrenosum; PGAR 100, target PG ulcer area reduction 100 (defined as complete skin re-epithelialization); SD, standard deviation.

†Five of the 10 patients with partial or non-response to treatment discontinued before week 26 due to an adverse event or other reason.

‡Of patients using concomitant corticosteroids.

Figure S1. Digital planimetry of Target PG ulcers during 52 weeks of treatment. (a) Patient 1; (b) Patient 2; (c) Patient 3.

(a)

**Screening**	**Baseline**	**Week 2**	**Week 6**	**Week 12**
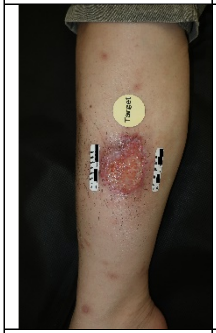	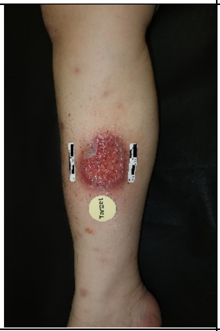	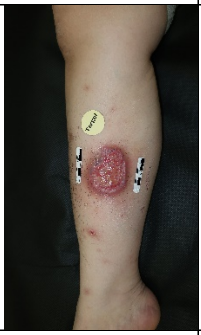	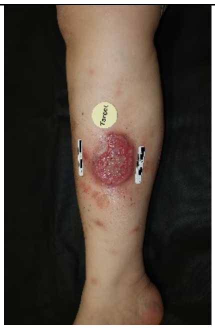	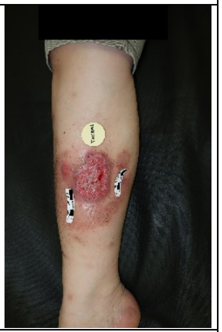
**Week 18**	**Week 26**	**Week 34**	**Week 42**	**Week 52**
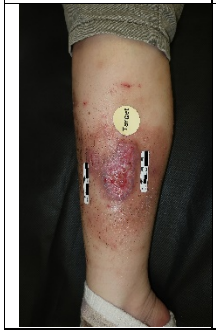	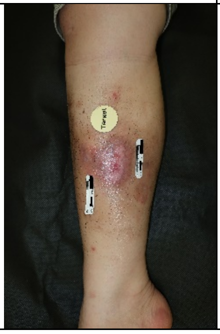	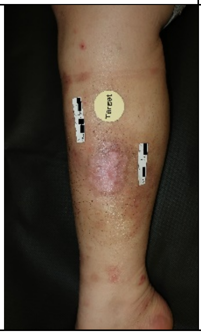	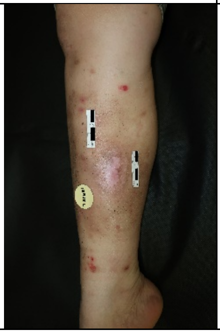	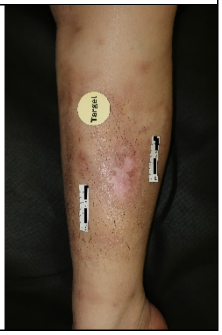

(b)

Screening	Baseline	Week 2	Week 6	Week 12
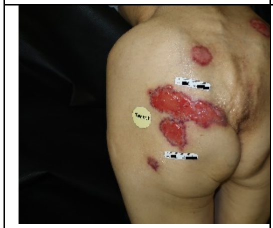	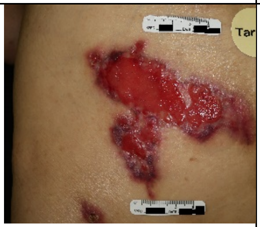	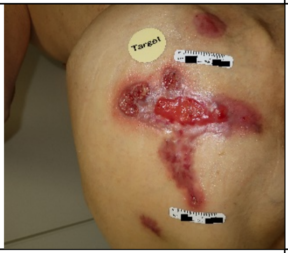	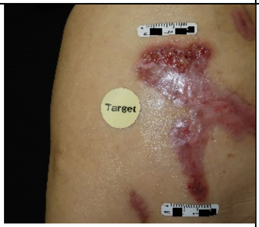	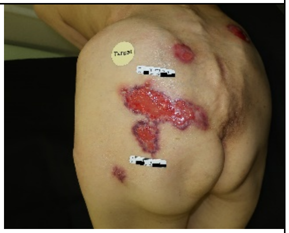
**Week 18**	**Week 26**	**Week 34**	**Week 42**	**Week 52**
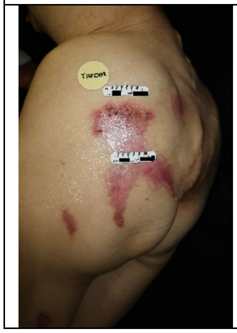	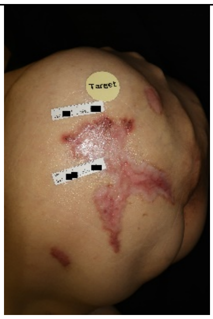	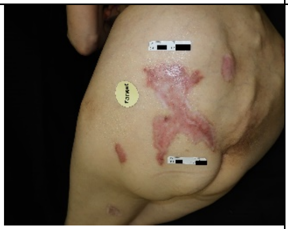	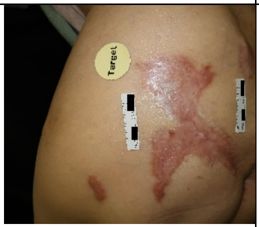	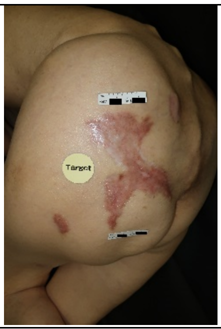

(c)

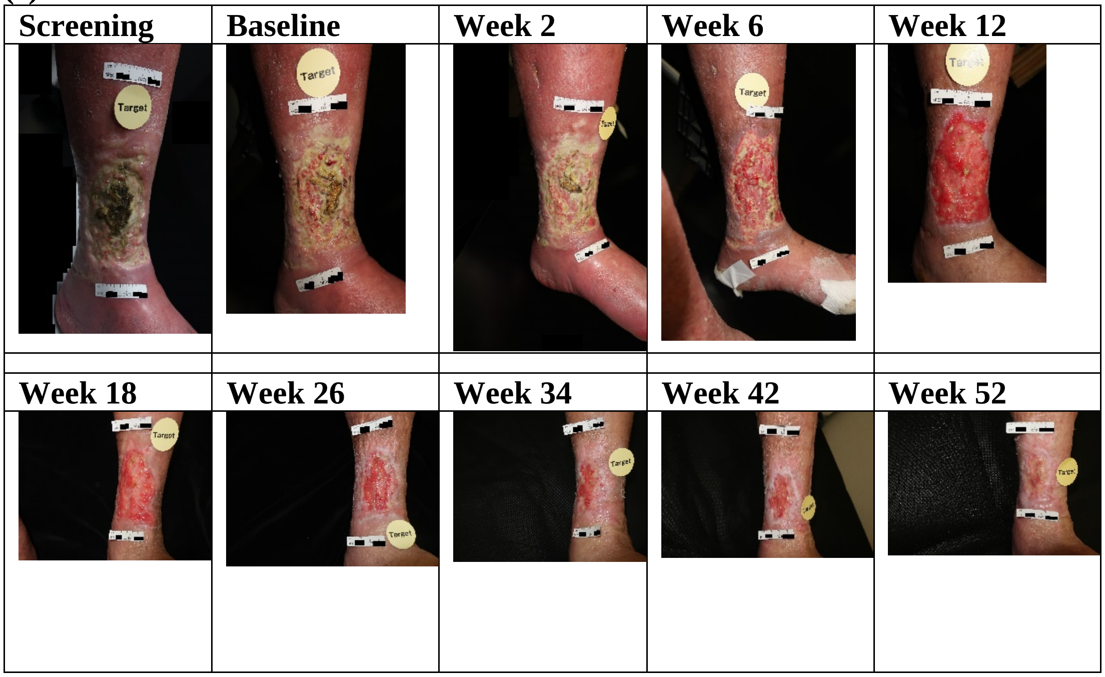
Screening
Baseline
Week 2
Week 6
Week 12
Week 18
Week 26
Week 34
Week 42
Week 52
Target
